# Ecological Adaptation in the Chemosensory Gene Repertoire of the Safflower Aphid, *Uroleucon gobonis*

**DOI:** 10.3390/ijms262311558

**Published:** 2025-11-28

**Authors:** Lanjie Xu, Minshan Sun, Wei Dong, Sufang An, Qing Yang, Hongqi Yang, Xiaohui Wu, Junping Feng, Zhengwei Tan, Yongliang Yu, Huizhen Liang

**Affiliations:** 1 Institute of Chinese Herbal Medicines, Henan Academy of Agricultural Sciences, Zhengzhou 450002, China; xulanjie18@163.com (L.X.); dongwei63@163.com (W.D.); ansufang888@163.com (S.A.); nzs3911@126.com (Q.Y.); yang13303833929@163.com (H.Y.); wuxiaohui8688@163.com (X.W.); junpingfeng123@163.com (J.F.); 290667278@126.com (Z.T.); 2 Provincial Key Laboratory of Conservation and Utilization of Traditional Chinese Medicine Resources in Henan, Zhengzhou 450002, China; 3 College of Plant Protection, Henan Agricultural University, Zhengzhou 450002, China; hnyxbio@163.com

**Keywords:** *Uroleucon gobonis*, chemosensory genes, stage-specific expression, tissue-specific expression, pest control targets

## Abstract

The red flower aphid (*Uroleucon gobonis*) is a significant agricultural pest causing damage via direct feeding and virus transmission. Chemical sensory proteins (CSPs) are critical for insecticide resistance, mediating the detection of semiochemicals or the sequestration of neuroactive insecticides. This study provides the first comprehensive identification and functional characterization of chemosensory gene families in *Uroleucon gobonis* to elucidate their roles in chemoperception and resistance. We conducted de novo transcriptome sequencing and assembly to identify chemosensory genes. Their phylogenetic relationships and structural motifs were analyzed. Developmental expression patterns were assessed via RNA-seq, and tissue-specific expression was validated using quantitative real-time PCR (qRT-PCR). We identified 40 chemosensory genes: 12 odorant-binding proteins (OBPs), 8 CSPs, 14 odorant receptors (ORs), and 6 gustatory receptors (GRs). Phylogenetic analysis revealed species-specific adaptations, including the absence of GR clades 2/4 and minimal representation in CSP Subgroup III. Structural motifs were highly conserved in ORs/OBPs but divergent in CSPs/GRs. RNA-seq identified 1896 differentially expressed genes (DEGs) between instars, including stage-specific regulation of *UgobCSP4*, *UgobCSP6*, *UgobOBP3*, and *UgobOBP10*. qRT-PCR confirmed extreme spatial expression, such as leg-specific *UgobCSP6* and antennae-specific *UgobOBP10*. These findings elucidate key molecular adaptations in chemosensory gene families governing perception and potential insecticide resistance in *Uroleucon gobonis*. The identified stage- and tissue-specific genes provide targets for developing species-specific pest control strategies.

## 1. Introduction

The safflower aphid, *Uroleucon gobonis*, constitutes a major pest of *Carthamus tinctorius*, significantly compromising both crop yield and medicinal quality through direct feeding damage and contamination of harvested materials [1,2]. Conventional insecticide control strategies face substantial limitations due to environmental persistence concerns and widespread resistance to major chemical classes including organophosphates, avermectins, carbamates, and pyrethroids [3,4,5].

Recent evidence indicates that the insect chemosensory system, beyond its canonical role in environmental perception, plays a critical part in insecticide resistance. Multidomain proteins—particularly chemosensory proteins (CSPs) and related ligand-binding factors—serve as key mediators of this resistance [6,7,8]. Their localization in sensory appendages and neural tissues enables the detection and sequestration of neuroactive compounds targeting key physiological sites: voltage-gated sodium channels, GABA-gated chloride channels, nicotinic acetylcholine receptors, and acetylcholinesterase (collectively accounting for ~85% of commercial insecticide targets) [6,7,8]. Structural analyses suggest these proteins either facilitate toxin entry via membrane transport or confer resistance through molecular sequestration, thereby modulating neurotoxic efficacy. Consequently, comprehensive characterization of their ligand-binding specificities and spatiotemporal expression patterns represents a critical avenue for elucidating novel resistance mechanisms [9,10]. This knowledge could enable the rational design of next-generation insecticides targeting dynamic protein–ligand interfaces rather than evolutionarily conserved neural targets—a strategic shift with significant potential to mitigate cross-resistance and environmental impact [11,12].

The insect chemosensory repertoire is composed of several large gene families. Olfactory receptors (ORs), members of the G protein-coupled receptor (GPCR) superfamily, feature seven transmembrane domains (7TMs) typically comprising 300–500 amino acids [13,14]. This receptor class operates through two functionally distinct units: the highly conserved odorant receptor co-receptor (Orco) and rapidly diversified ligand-binding receptors [15]. These subunits assemble as heteromeric complexes where Orco forms the ion channel core while specific ORs directly bind volatile ligands. Phylogenetic analyses reveal that the OR family originated from gustatory receptors (GRs) at the base of insect radiation, with accelerated diversification coinciding with chemosensory niche specialization [16,17].

Odorant-binding proteins (OBPs; 14–17 kDa) feature conserved cysteine-stabilized ligand-binding cavities and are classified into Classic (6C), Minus-C (4C), Plus-C (≥8C), and Atypical (0–3C) subtypes [18,19,20,21]. Following their discovery in *Antheraea polyphemus*, lineage-specific expansions across insects underpin semiochemical recognition. Chemosensory proteins (CSPs; 10–12 kDa) exhibit broader functionality—from odorant solubilization (*Periplaneta americana* P10) to insecticide sequestration (*Cydia pomonella* CSP1) [12,22,23].

Gustatory receptors (GRs) represent an evolutionarily older receptor lineage [24,25,26,27]. They retain the 7TM topology but have diversified into four functionally distinct subtypes: fructose-specific receptors, non-fructose sugar receptors, CO_2_ sensors, and bitter receptors (T2Rs) [28,29,30]. Bitter receptors serve as defense sensors by recognizing phytotoxins to initiate avoidance behaviors, while CO_2_ receptors drive host-seeking in vector mosquitoes. Functional studies indicate GRs likely operate as ligand-gated ion channels that directly depolarize neurons—a mechanism electrophysiologically validated in *Drosophila* [31,32,33,34]. The evolutionary linkage between core chemosensory functions (odorant transport by OBPs/CSPs and recognition by ORs/GRs) and adaptive traits like insecticide resistance in *Uroleucon gobonis* underscores potential functional trade-offs governing ecological interactions and xenobiotic defense [11,12].

However, despite their agricultural importance, a comprehensive identification and functional characterization of these critical chemosensory gene families has not been conducted for *Uroleucon gobonis*. This gap limits our understanding of the molecular basis of its chemoperception and potential resistance mechanisms. Therefore, the aim of this study was to perform a genome-wide identification and characterization of the OBPs, CSPs, ORs, and GRs in *Uroleucon gobonis* from a de novo assembled transcriptome. We analyzed their phylogenetic relationships, conserved structural motifs, and expression profiles across different developmental stages and tissues. Our findings provide foundational insights into the molecular basis of chemosensation in this pest and identify potential target genes for developing novel, species-specific pest management strategies.

## 2. Results

### 2.1. Transcriptome Assembly and Assessment

To obtain a comprehensive reference transcriptome, pooled RNA-Seq libraries were constructed using RNA samples from six distinct developmental stages of *Uroleucon gobonis*, each with three biological replicates. A total of 122.18 GB of clean data was generated, with each sample yielding at least 6.33 GB. The percentage of Q30 bases was 92.53% or higher across all samples. To ensure the reliability of transcriptomic analyses, the quality of the sequencing libraries was rigorously assessed from three perspectives. First, the randomness of mRNA fragmentation and potential mRNA degradation were evaluated by examining the distribution of insert fragments across unigenes. Second, the dispersion of insert fragment lengths was assessed by plotting their size distribution. Third, library capacity and the sufficiency of reads mapping to the unigene library (Mapped Reads) were evaluated using saturation curves. The randomness of mRNA fragmentation was further verified through simulation. This was based on the positional distribution of Mapped Reads along unigenes. The results demonstrated that the positional distribution curve of Mapped Reads along mRNA transcripts exhibited a shallow slope in the central region, indicating an absence of significant degradation (Figure S1). Statistical analysis of insert fragments revealed a major peak between 280 bp and 400 bp, which was within the target range, and a narrow peak shape, indicating minimal length dispersion and normal fragment size selection. Saturation curve analysis for quantification reflected the sequencing depth required for accurate gene expression quantification. Highly expressed genes are readily quantified accurately, whereas accurate quantification of lowly expressed genes requires greater sequencing depth. The attainment of saturation in these curves confirmed that the sequencing data volume was sufficient to meet quantitative requirements.

Building upon these high-quality sequencing data, de novo assembly using Trinity generated 49,483 transcripts and 22,341 unigenes. The assembly exhibited high completeness, as reflected by N50 values of 3147 bp for transcripts and 2849 bp for unigenes. Analysis of length distributions revealed distinct patterns: within the 300–500 bp range, there were 7349 transcripts (14.85% of total transcripts) and 5692 unigenes (25.48% of total unigenes) (Figure S2). Notably, transcripts consistently constituted a higher proportion than unigenes by approximately 5% in both the 500–1000 bp and 1000–2000 bp length intervals. The most pronounced difference was observed in sequences exceeding 2000 bp, where transcripts numbered 20,262 (40.95% of total transcripts), significantly surpassing the 6659 unigenes (29.81% of total unigenes) in this category. Collectively, these results demonstrate the high quality of our sequencing data and indicate the presence of substantial transcript isoform diversity within the *Uroleucon gobonis* transcriptome.

### 2.2. Functional Annotation and SSR Identification

Functional annotation of the assembled unigenes was performed using BLAST (Version 2.17.0) against the NR, Swiss-Prot, GO, COG, KOG, and KEGG databases. Following amino acid sequence prediction, unigenes were further analyzed against the Pfam database using HMMER. Applying a stringent E-value threshold of ≤10^−5^, a total of 13,575 unigenes were successfully annotated. Among the databases, NR provided the highest annotation coverage (13,405 unigenes), while GO (Gene Ontology) yielded the fewest annotations (4046 unigenes). GO enrichment analysis of these annotated genes revealed distinct functional distributions. The majority of genes were significantly enriched in Biological Process terms, primarily associated with cellular process, metabolic process, and biological regulation. Cellular Component terms represented the second most abundant category, dominated by annotations related to cell, cell part, and organelle. Conversely, Molecular Function terms exhibited the lowest enrichment, with predominant roles in binding, catalytic activity, and transporter activity (Figure S3).

**Figure 1:**
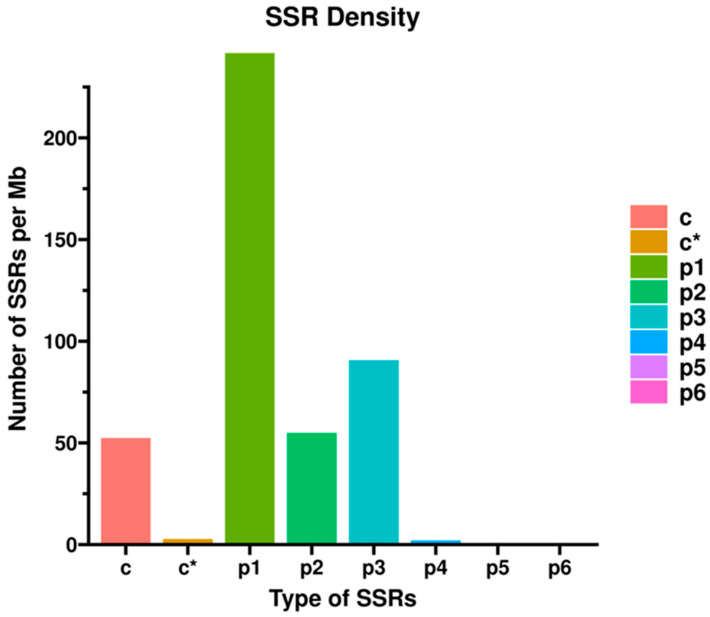
Statistical distribution of identified SSR types. x-axis: SSR type; y-axis: number of corresponding SSR motifs per Mb of sequence. c, tandem repeats without embedded complex repeats; c*, tandem repeats with embedded complex repeats; p1, single-base repeats; p2, two-base repeats; p3, three-base repeats; p4, four-base repeats; p5, five-base repeats; p6, six-base repeats.

Furthermore, analysis of the unigene sequences identified six distinct types of Simple Sequence Repeats (SSRs): mono-nucleotide, di-nucleotide, tri-nucleotide, tetra-nucleotide, penta-nucleotide, and hexa-nucleotide repeats. The results revealed that p1-type SSRs (mono-nucleotide repeats) exhibited the highest density, with the greatest number of occurrences per megabase (Mb) of sequence. In contrast, the abundance of p5 (penta-nucleotide) and p6 (hexa-nucleotide) SSRs was negligible, approaching zero (Figure 1).

### 2.3. Characteristics of Chemosensory Genes in Uroleucon gobonis

Building upon the transcriptome assembly, we identified chemosensory-related protein genes from the de novo transcriptome of *Uroleucon gobonis*. A total of 40 candidate genes were characterized, comprising 12 odorant binding proteins (OBPs), 8 chemosensory proteins (CSPs), 14 olfactory receptors (ORs), and 6 gustatory receptors (GRs); detailed annotations are provided in Table S1. Comprehensive characterization of these *Uroleucon gobonis* chemosensory-related proteins included analyses of gene structure metrics, protein properties (amino acid length, isoelectric point, molecular weight), and structural features (predicted subcellular localization). As summarized in Table S1, the encoded proteins exhibited substantial size variation, ranging from 114 amino acid residues (UgobCSP7) to 952 aa (UgobOR13). The pI values spanned a broad range from 4.72 (UgobCSP8) to 9.91 (UgobOBP10), while MWs varied between 12.77 kDa (UgobCSP7) and 81.49 kDa (UgobOR13). Subcellular localization predictions consistently indicated that all identified chemosensory-related proteins were distributed across multiple compartments, including the cytoplasm, plasma membrane, and extracellular space.

### 2.4. Phylogenetic Analysis of Chemosensory Genes Family in Uroleucon gobonis

**Figure 2:**
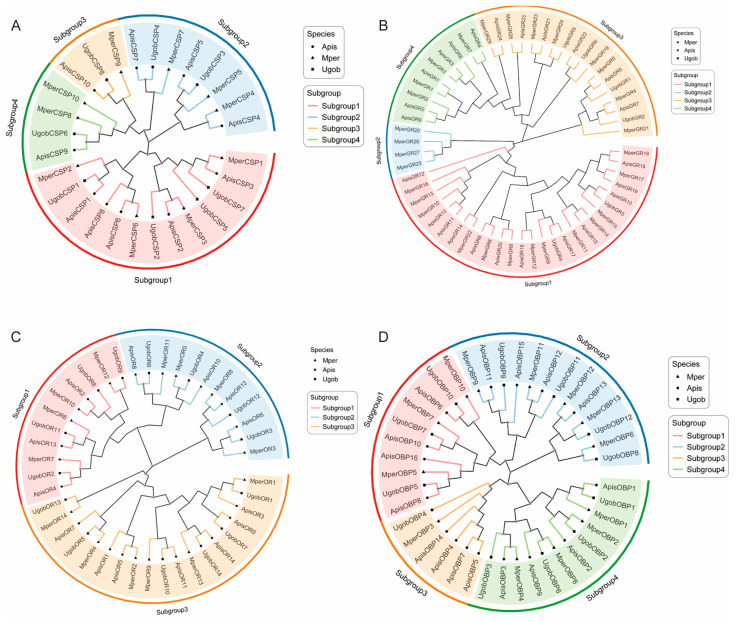
Phylogenetic analysis of chemosensory gene families in *Uroleucon gobonis*. (**A**) Phylogenetic tree of CSPs. (**B**) Phylogenetic tree of GR proteins. (**C**) Phylogenetic tree of OR proteins. (**D**) Phylogenetic tree of OBPs. Trees were constructed using the maximum likelihood method. Sequences from *Acyrthosiphon pisum* (*Apis*), *Myzus persicae* (*Mper*), and *Uroleucon gobonis* (*Ugob*) are included.

To evaluate the evolutionary relationships of chemosensory genes in *Uroleucon gobonis*, maximum likelihood (ML) phylogenetic trees were constructed for four chemosensory protein gene families using sequences from three aphid species. Consistent with established classification schemes for CSP families in other organisms, the CSP members were classified into four major subclasses: Subclass I contained 13 genes, with *UgobCSPs* representing 30.8% (n = 4); Subclass II comprised 8 genes, including only 2 *UgobCSP* members; Subclass III was the smallest group (3 genes), containing one representative from each of the three aphid species; and Subclass IV contained 4 genes, with a single *UgobCSP* represented (Figure 2A).

Phylogenetic analysis revealed distinct evolutionary patterns across chemoreceptor families. Gustatory receptors (GRs) formed four major clades, notably lacking *Uroleucon gobonis* representatives in Clade 2 and Clade 4, suggesting a potential loss of these functional orthologs in this species (Figure 2B). Regarding olfactory receptors (ORs), we identified 14 candidate genes in *Uroleucon gobonis*. Intriguingly, each of the three studied aphid species possessed precisely 14 OR candidates. The OR phylogeny resolved into three primary subgroups: Subgroup III contained the highest number of ORs (n = 19), with one aphid species contributing seven members and the other two species each possessing six (Figure 2C). For odorant binding proteins (OBPs), 12 genes were identified in *Uroleucon gobonis*, clustering into four subgroups. Strikingly, Subgroup III contained the fewest members, indicating a potential functional deficit for this OBP subclass in *Uroleucon gobonis* (Figure 2D).

### 2.5. Structure Analysis of Uroleucon gobonis Chemosensory Genes

**Figure 3:**
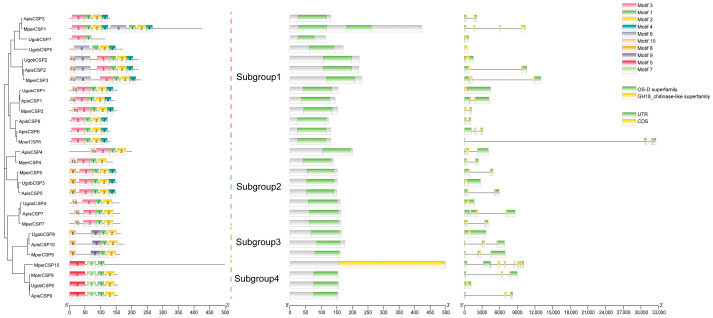
Analysis of phylogenetic relationships, protein domains, and gene structures of CSPs. Conserved protein motifs in the amino acid sequences of CSPs were analyzed using the MEME suite. Visualization of gene structures for CSPs, including exon–intron organization and untranslated regions (UTRs). Identification and characterization of functional domains within CSPs.

To investigate potential functional divergence across subgroups, we performed structural motif analysis of each gene family. Within CSPs, 10 conserved motifs were identified. Motifs 1, 2, and 3 were present in nearly all subgroups. Subgroup-specific motifs were observed: motif 6 was predominantly unique to Subgroup I, while motif 8 was characteristic of Subgroups II and III, though their motif arrangements differed (Subgroup III consistently exhibited an 8-9-1-2 motif order). Subgroup IV displayed the most distinct profile, featuring two unique motifs and a conserved 5-7-1-2 arrangement (Figure 3). Parallel analyses of other families revealed intriguing patterns. In GRs, Subgroup I exhibited greater complexity, typically containing motifs 9-8-5-7-2-3-1-4, whereas other subgroups generally possessed fewer than six motifs (Figure S4). OBP subgroups demonstrated high motif conservation, all sharing motif 1 and exhibiting similar motif numbers (Figure S5). ORs showed the highest sequence conservation across subgroups, with nearly all genes containing the core motif combination 1-5-4-6-7; motifs 3 and 9 served as key distinguishing features (Figure S6). Collectively, these results indicate distinct evolutionary constraints: OR and OBP families exhibit high sequence conservation suggestive of conserved functions, whereas CSPs and GRs display significant structural divergence across subgroups, likely contributing to functional diversification.

### 2.6. RNA-Seq and qRT-PCR Profiling of Gene Expression Patterns

**Figure 4:**
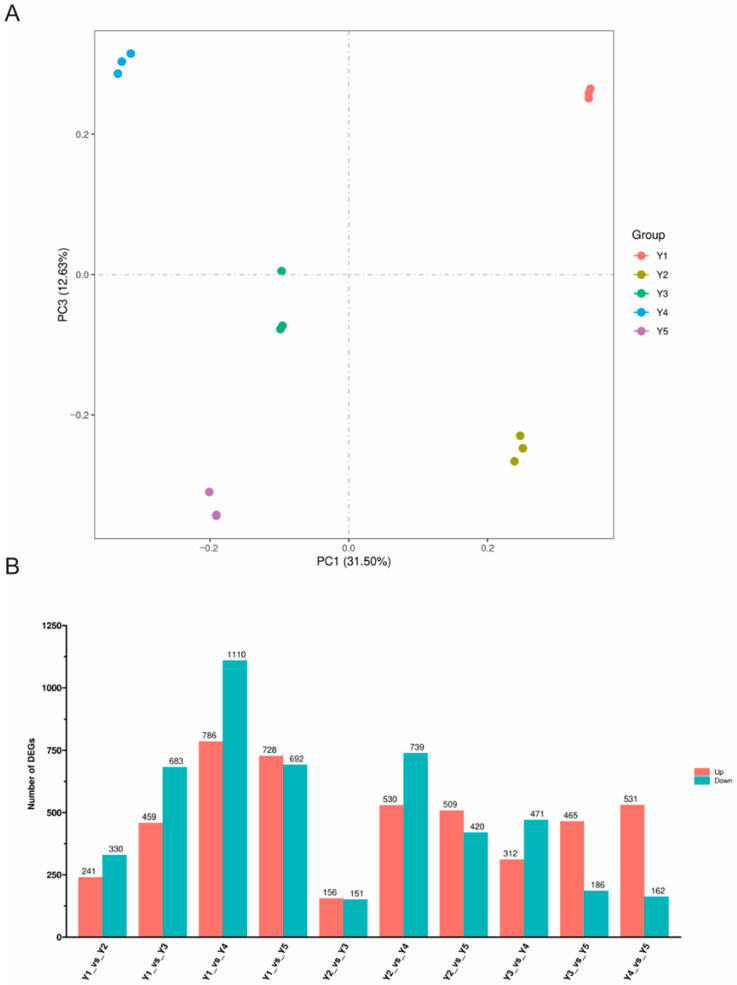
RNA-seq analysis results. (**A**) Principal component analysis (PCA) of sample transcriptomic profiles. (**B**) Summary of differentially expressed genes (DEGs).

To investigate the expression dynamics of these gene families across *Uroleucon gobonis* development, RNA-seq was performed on samples representing six key stages: 1st instar, 2nd instar, 3rd instar, 4th instar, and adult, with three biological replicates per stage. Following quality control and quantification of the developmental transcriptome, Principal Component Analysis (PCA) revealed high reproducibility, as replicates clustered tightly together within each stage (Figure 4A and Table S2). Furthermore, distinct separation between developmental stages along the principal components indicated significant transcriptomic divergence across the life cycle. Differential expression analysis identified pronounced transcriptional changes between stages. The most substantial differences occurred between the 1st and 4th instars, with 786 significantly upregulated and 1110 downregulated genes, suggesting major physiological transformations during this period (Figure 4B). In contrast, minimal transcriptional divergence was observed between the 2nd and 3rd instars, exhibiting only 156 upregulated and 151 downregulated genes. Screening the differentially expressed genes (DEGs) for members of the four characterized chemosensory families identified four significantly regulated candidates: *UgobCSP4* (*DN3734*), *UgobCSP6* (*DN3991*), *UgobOBP10* (*DN199*), and *UgobOBP3* (*DN16170*). This finding highlights specific chemosensory genes potentially involved in the substantial physiological adaptations occurring during aphid development.

**Figure 5:**
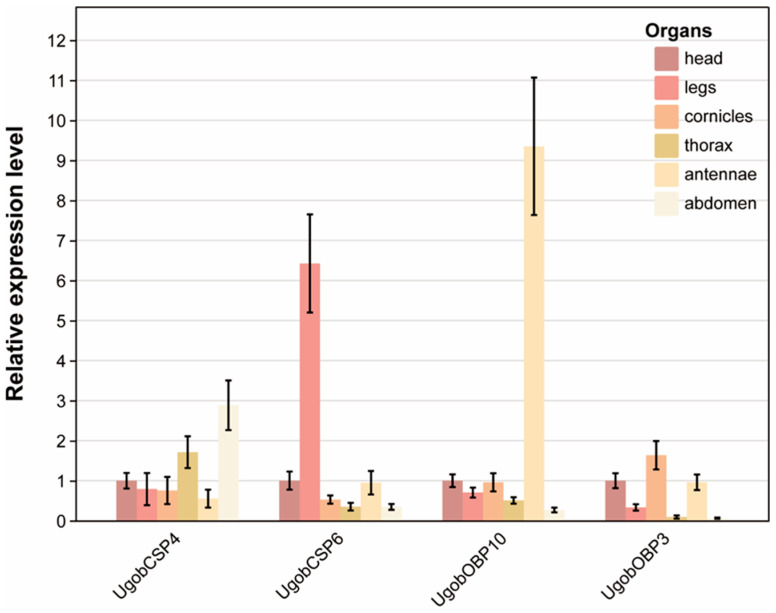
Tissue-specific expression profiles of four significantly differentially expressed chemosensory genes validated by qRT-PCR. Bars represent mean relative expression levels (±standard deviation, SD) of three biological replicates, normalized to reference genes and calculated using the 2^−ΔΔCt^ method.

To elucidate the tissue-specific expression patterns of these four candidate genes, we performed qRT-PCR analysis on six dissected tissues from mature *Uroleucon gobonis*: head, legs, cornicles, thorax, antennae, and abdomen (Table S3 and Figure S7). Consistent with their differential expression during development, these genes exhibited distinct spatial expression profiles (Figure 5). *UgobCSP4* displayed significantly higher expression in the abdomen compared to other tissues, with its lowest expression level observed in the cornicles (less than one-fifth of the abdominal expression). The expression pattern of *UgobCSP6* was more restricted, showing exclusive and markedly elevated expression in the legs, far exceeding levels in any other tissue, suggesting a leg-specific functional role. *UgobOBP10* exhibited a similarly restricted pattern, with exceptionally high expression confined solely to the antennae. Interestingly, *UgobOBP3* demonstrated relatively high and consistent expression levels across three tissues: head, cornicles, and antennae, while exhibiting significantly lower expression in the thorax, legs, and abdomen.

## 3. Discussion

Our genome-wide identification of chemosensory genes in *Uroleucon gobonis* not only provides a catalog of potential molecular players in perception and defense but also reveals striking evolutionary adaptations shaped by its specialized ecological niche—the safflower (*Carthamus tinctorius*) chemosensory environment. The observed gene family contractions, structural innovations, and tissue-specific expression patterns collectively suggest a fine-tuned chemosensory system optimized for a monocultural host, with significant implications for insecticide response.

The absence of GR clades 2/4 (Figure 2B) aligns with gene family contractions observed in host-specialized aphids like *Schlechtendalia chinensis* [35,36,37], suggesting convergent simplification of chemosensory repertoires under monocultural selection. The leg-specific expression of *UgobCSP6* (Figure 5) is particularly revealing. As the legs are the primary appendages contacting plant surfaces and insecticide residues, this spatial restriction strongly suggests a role in the initial encounter and sequestration of environmental chemicals. The functional parallel with *RpCSP6*, which binds and sequesters the insecticide deltamethrin [10,38,39], provides a compelling hypothesis: *UgobCSP6* may serve as a first-line defense protein in *Uroleucon gobonis*, binding similar pyrethroid insecticides or specific host plant secondary metabolites encountered during locomotion. The classical olfactory roles are exemplified by *UgobOBP10* and *UgobOBP3*. The strict antennal-specificity of *UgobOBP10* (Figure 5), coupled with its homology to an EBF-binding OBP, marks it as a key candidate for sensing the aphid alarm pheromone. Meanwhile, the broad expression of *UgobOBP3* in the head, cornicles, and antennae suggests a dual role. Its presence in the cornicles, which emit alarm pheromone, hints at a potential, previously unconsidered role in the release or “scavenging” of semiochemicals, while its expression in olfactory appendages supports a complementary function in general odorant perception [40,41].

Beyond these highly tissue-specific genes, the differential expression of *UgobOBP3* and *UgobCSP4* also suggests distinct physiological roles. Conversely, the abdominal dominance of *UgobCSP4* expression (Figure 5) points towards a role in internal physiological processes. The abdomen houses the digestive and reproductive systems, as well as key detoxification organs. The review by Rihani et al. documents numerous examples of OBPs and CSPs expressed in non-sensory tissues, including the digestive tract and reproductive organs, where they are hypothesized to solubilize and transport dietary lipids, hormones, or other hydrophobic compounds [40]. Therefore, *UgobCSP4* is unlikely to be involved in external odorant detection but may instead function in the internal transport of ingested plant compounds (e.g., safflower-specific metabolites), in insecticide detoxification post-ingestion, or even in reproductive physiology through the handling of hydrophobic signaling molecules [38,39]. Conversely, *UgobOBP3* demonstrated relatively high and consistent expression across the head, cornicles, and antennae. Its presence in the cornicles—organs known for alarm pheromone emission—hints at a potential involvement in the detection or release of semiochemicals, while its expression in the head and antennae may indicate a complementary role in general odorant perception.

The most compelling evidence for ecological specialization comes from the conspicuous absence of GR clades 2/4 (Figure 2B). In generalist aphids like *Acyrthosiphon pisum*, clade 2 GRs function as bitter receptors that trigger avoidance behaviors upon sensing non-host plant phytotoxins [42,43]. The loss of these receptors in *Uroleucon gobonis* likely represents an adaptive evolutionary step, freeing the aphid from repulsive signals that might otherwise deter it from its exclusive host, safflower. This genomic streamlining, converging with observations in other host-specialized aphids [35,36,37,44,45], underscores a trade-off: refined host fidelity at the cost of behavioral flexibility. This concept of chemosensory repertoire simplification underpinning host specialization is a key theme in insect chemical ecology, as highlighted in the recent synthesis by Rihani et al. [40].

Similarly, the minimal representation in CSP Subgroup III (Figure 2A)—a clade implicated in broad-spectrum xenobiotic sequestration in moths like *Cydia pomonella* [6,46]—further reflects the unique chemical pressures faced by *Uroleucon gobonis*. Long-term specialization on a single host plant likely reduces encounters with a wide array of plant-derived insecticides, diminishing the evolutionary pressure to maintain a diverse arsenal of detoxification proteins like CSPs. Instead, as we discuss below, the CSP repertoire of *Uroleucon gobonis* appears to have been reconfigured for more specific tasks.

It is important to note that our expression analysis did not distinguish between sexes, as the focus was on developmental stages and early instars are asexual. However, sexual dimorphism in chemosensory gene expression has been documented in other aphid species (e.g., *Acyrthosiphon pisum*), where distinct ecological roles (e.g., host-finding in females vs. mate-seeking in males) can drive differential gene regulation [41,47]. Therefore, future investigations involving sex-specific sampling of adult *Uroleucon gobonis* would be valuable to elucidate any potential dimorphic expression patterns, which could further refine the development of behavior-based control strategies.

## 4. Materials and Methods

### 4.1. De Novo Transcriptome Assembly Based on RNA-Seq

High-throughput sequencing was performed on the DNBSEQ-T7RS platform to obtain raw reads. De novo transcriptome assembly was carried out with the Trinity software (Version 2.8.3) suite [48]. Prior to assembly, quality filtering of the raw reads was conducted using Trimmomatic with the following parameters: LEADING:30, TRAILING:30, SLIDINGWINDOW:4:20, and MINLEN:50 [49]. Clean reads from the two pooled libraries were merged and then digitally normalized using Trinity’s default settings to reduce runtime and memory consumption. Assembly with Trinity was executed using a k-mer size of 25 and a minimum k-mer coverage of 2. The resulting contigs were subsequently processed with the TGI Clustering Tool to address alternative splicing and remove redundant sequences.

### 4.2. De Novo Transcriptome Annotation

Open reading frames (ORFs) were extracted with TransDecoder (retaining ≥30-aa peptides) (https://github.com/TransDecoder/TransDecoder; accessed on 10 May 2025) and additionally predicted by GeneMarkS-T. The two ORF sets were merged with CD-HIT to remove redundancy. Homology searches were performed against NR, Swiss-Prot and RefSeq protein databases (BLASTP, e-value ≤ 10^−5^) and against NT/RefSeq nucleotide databases (BLASTX/BLASTN, e-value ≤ 10^−10^), retaining the top hit per transcript. Conserved domains were identified with Pfam and InterProScan; signal peptides and transmembrane helices were predicted using SignalP (Version 6.0) and TMHMM (Version 2.0), respectively. Functional annotations and GO classifications were integrated in Trinotate, while KEGG pathway assignments were generated with GhostKOALA.

### 4.3. Identification of Chemosensory Gene Families in Uroleucon gobonis

All protein-coding sequences of the OBPs, GRs, ORs, and CSPs families in the *Uroleucon gobonis* genome were searched using HMMER (http://www.cbs.dtu.dk/services/TMHMM/; version 3.0; accessed on 10 May 2025). The annotation table, obtained by integrating three approaches (de novo prediction, homology search, and transcript-based assembly), was used to identify gene families: the GR gene family was identified using the hidden Markov model (HMM) profile from Pfam (PF08395), while CSP, OR, and OBP were identified using trained HMM profiles. All sequences were validated against the NCBI Conserved Domain Database (NCBI-CDD) (e-value = 10^−5^), and those lacking conserved domains were discarded. Sequences verified by both methods were considered potential functional genes.

### 4.4. Sequence Alignment and Phylogenetic Analysis

Full-length amino acid sequences of OBPs, GRs, ORs and CSPs from *Uroleucon gobonis*, *Acyrthosiphon pisum*, and *Myzus persicae* were aligned with ClustalW (https://www.genome.jp/tools-bin/clustalw; version 2.1; accessed on 10 May 2025) using default parameters. Maximum likelihood (ML) phylogenetic trees were constructed in MEGAX with 1000 bootstrap replicates under the Poisson model, and visualized using EvolView (https://www.evolgenius.info/evolview version 1.0; accessed on 10 May 2025) [50,51].

### 4.5. Analysis of the Conserved Protein Motifs and Gene Structure

Gene structures (exons/introns) of chemosensory gene families were visualized using GSDS (http://gsds.gao-lab.org/; version 2.0; accessed on 10 May 2025). Conserved protein motifs were identified via MEME with parameters: motif width 6–200 residues, maximum motifs = 20, distribution = 0/1. Results were integrated with phylogenetic trees and *Uroleucon gobonis* GFF3 data using TBtools (V1.098) [52].

### 4.6. Expression Profile of Chemosensory Genes

Clean RNA-seq reads were aligned to the de novo assembled transcriptome using Bowtie2 (Version 2.5.4) with default parameters [53]. To ensure alignment accuracy, reads with mapping quality scores < 20 were discarded. Transcript abundance was then estimated using RSEM, which effectively handles ambiguously mapped reads across transcript isoforms (https://github.com/deweylab/RSEM; v1.3.1; accessed on 10 May 2025). Expression levels were normalized as Transcripts Per Million (TPM) and Fragments Per Kilobase of transcript per Million mapped reads (FPKM) to enable cross-sample comparisons.

For differential gene expression analysis, the read count matrix generated by RSEM was imported into edgeR and DESeq2 [54]. Low-abundance transcripts with a mean TPM < 1 across all samples were filtered out to reduce noise. Normalization factors were calculated using the trimmed mean of M-values (TMM) method in edgeR and the median-of-ratios method in DESeq2. Generalized linear models (GLMs) with quasi-likelihood tests (edgeR) or Wald tests (DESeq2) were applied to identify significantly differentially expressed genes (DEGs), defined as those with |log_2_(fold change)| ≥ 1 and a false discovery rate (FDR)-adjusted *p*-value < 0.05.

### 4.7. RNA Extraction and qRT-PCR Analysis

Briefly, tissues were homogenized in 200 μL RNAiso Plus with a tissue grinder (60 Hz, 3 min), and the lysate was centrifuged at 12,000× *g* for 5 min at 4 °C. The aqueous phase was transferred to a new RNase-free tube, mixed with 0.2 volumes of chloroform, and centrifuged again at 12,000× *g* for 15 min at 4 °C. RNA was precipitated by adding an equal volume of isopropanol, incubated at room temperature for 10 min, and pelleted by centrifugation (12,000× *g*, 10 min, 4 °C). The RNA pellet was washed with 75% ethanol, air-dried under sterile conditions, and dissolved in RNase-free H_2_O. RNA concentration and quality were assessed using a NanoDrop spectrophotometer (Thermo Fisher Scientific; located in Shanghai, China) after 10-fold dilution in DEPC-treated water, while integrity was verified by 1.5% agarose gel electrophoresis (stained with nucleic acid dye) under UV light, where three distinct bands (28S, 18S, and 5S rRNA) indicated intact RNA. For cDNA synthesis, 1 μg of total RNA was treated with 4× gDNA Wiper Mix (Vazyme, located in Shanghai, China) to eliminate genomic DNA contamination, followed by reverse transcription using 5× HiScript III Qrt SuperMix (Vazyme) according to the manufacturer’s protocol (42 °C for 2 min, 37 °C for 15 min, and 85 °C for 5 s). The resulting cDNA was diluted 2-fold with ddH_2_O. Quantitative real-time PCR (qRT-PCR) was performed using Hieff qPCR SYBR Green Master Mix (No Rox; Vazyme) with gene-specific primers (0.5 μL each) in a 20 μL reaction volume. The primer information is shown in Supplementary Table S3. The cycling program consisted of an initial denaturation at 95 °C for 5 min, followed by 40 cycles of 95 °C for 20 s, 60 °C for 20 s, and 72 °C for 20 s, with a final extension at 72 °C for 20 s. Relative gene expression was calculated using the 2^−ΔΔCt^ method, normalized to reference genes. All steps were conducted on ice using RNase-free consumables to prevent degradation.

## 5. Conclusions

We decipher the chemosensory landscape of *Uroleucon gobonis* through integrated omics approaches. Key conclusions include the following: (1) Lineage-specific gene loss (GR clades 2/4) reflects ecological adaptation to safflower monoculture. (2) Structural divergence in CSPs/GRs correlates with functional plasticity in insecticide sequestration. (3) Spatiotemporal regulation of *UgobCSP6* and *UgobOBP10* identifies tissue-specific targets for precision pest management. These findings establish a foundation for developing species-specific control agents that disrupt chemoperception-mediated resistance mechanisms. Collectively, our study moves beyond a mere gene catalog by demonstrating how the integrated evolution of gene repertoire, protein structure, and spatiotemporal expression equips *Uroleucon gobonis* to thrive in its specific chemosensory environment, thereby establishing a foundation.

## Data Availability

All the raw RNA-seq data in this study are publicly available in the NCBl BioProject with the accession PRJNA1291393. The original contributions presented in this study are included in the article/Supplementary Material. Further inquiries can be directed to the corresponding authors.

## Supplementary Materials

The following supporting information can be downloaded at: https://www.mdpi.com/article/10.3390/ijms262311558/s1.
