# Supplementary material for: Ecological Adaptation in the Chemosensory Gene Repertoire of the Safflower Aphid, Uroleucon gobonis
Source: Int J Mol Sci. 2025 Nov 28;26(23):11558. doi: 10.3390/ijms262311558 (PMC12692544; doi:10.3390/ijms262311558)
Supplement: Supplementary file 1 [file ijms-26-11558-s001.zip › ijms-3814344-supplementary.pdf]

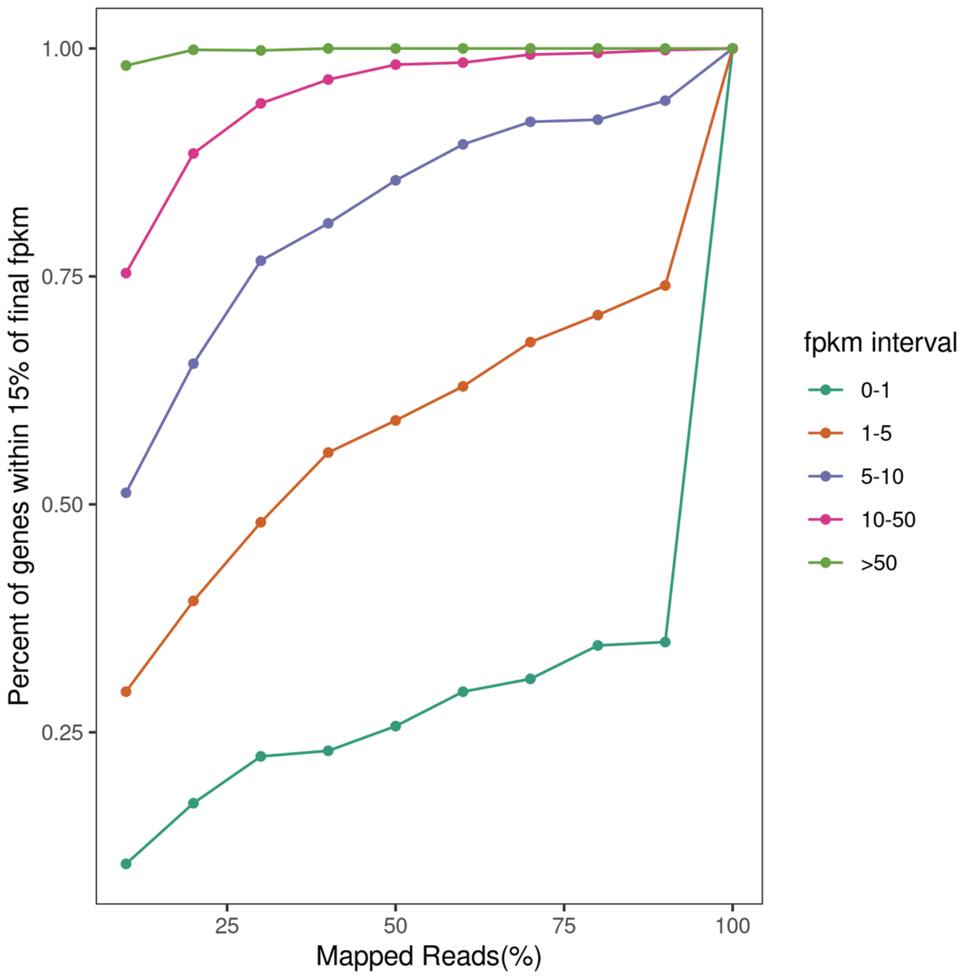

Figure S1: Transcriptome sequencing data saturation curve

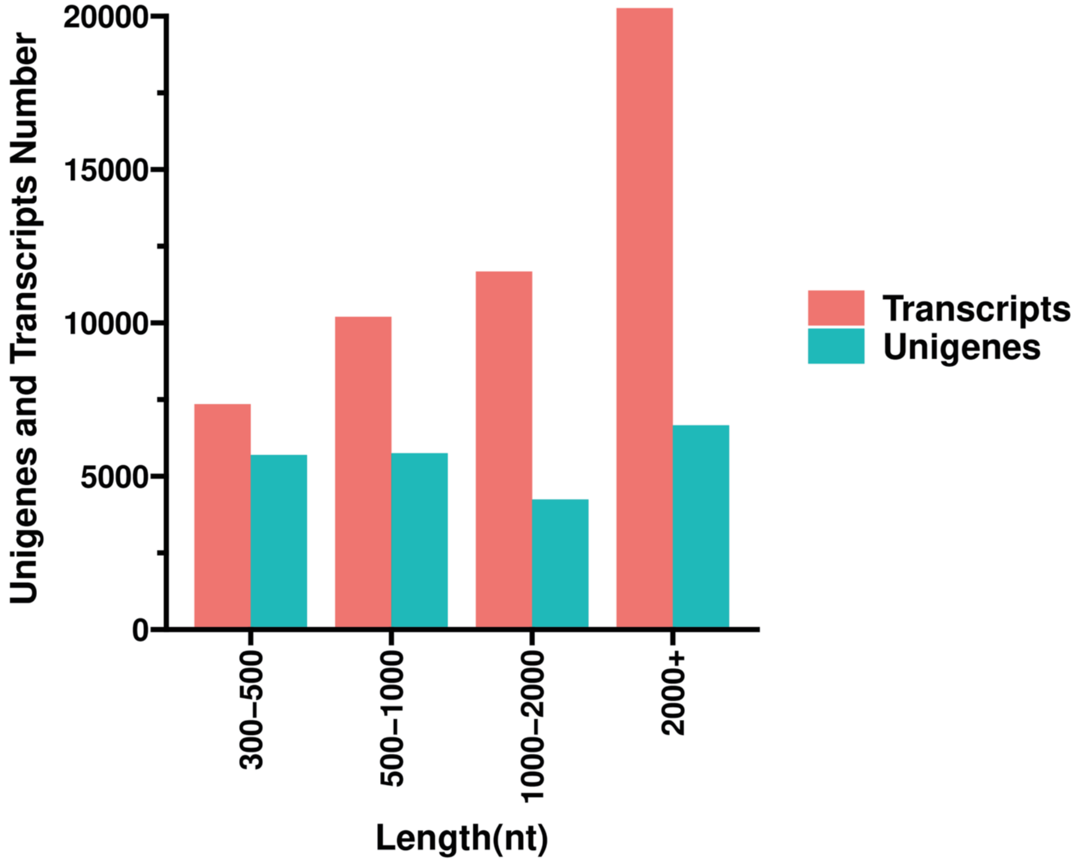

Figure S2: Distribution of unigene lengths

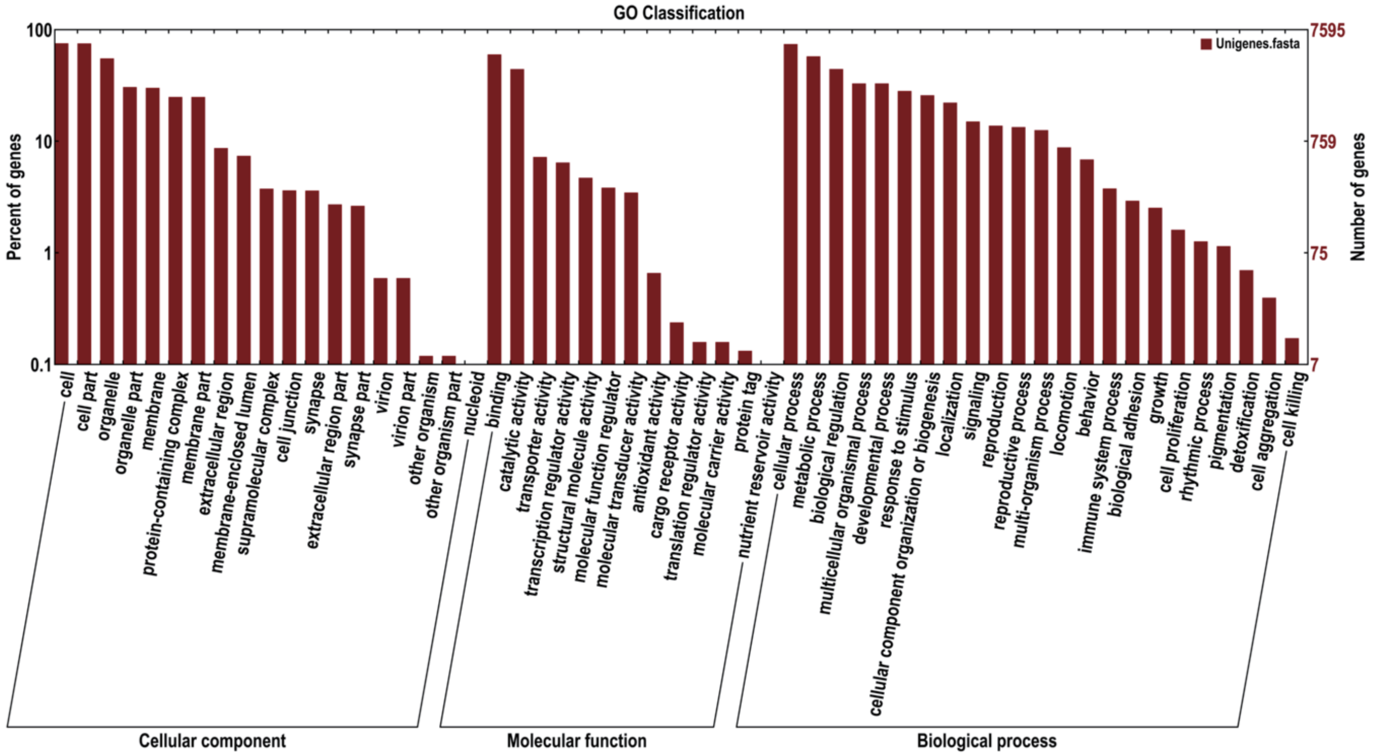

Figure S3: GO enrichment analysis of expressed genes versus all genes

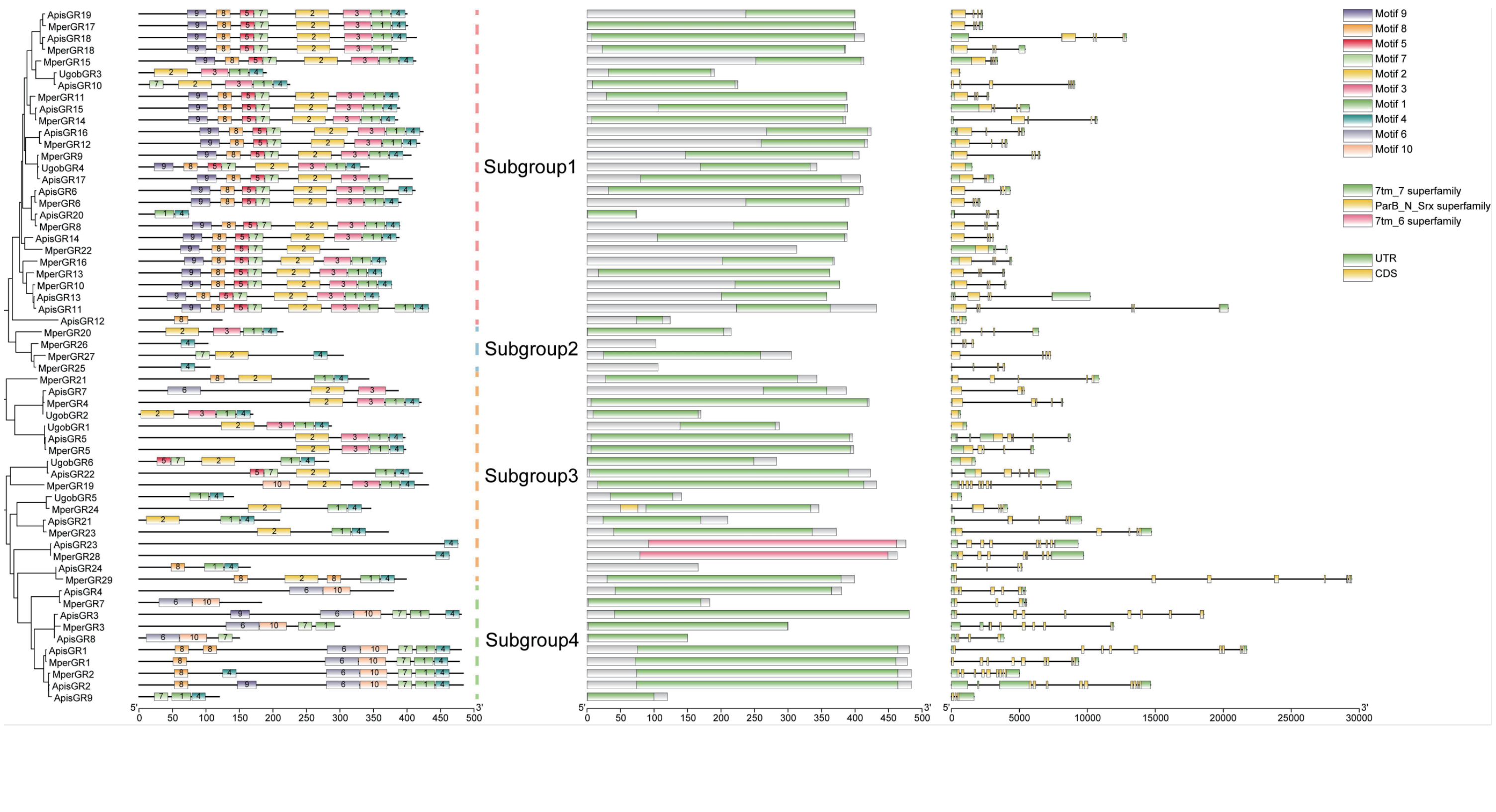

Figure S4: Analysis of phylogenetic relationships, protein domains, and gene structures of GRs

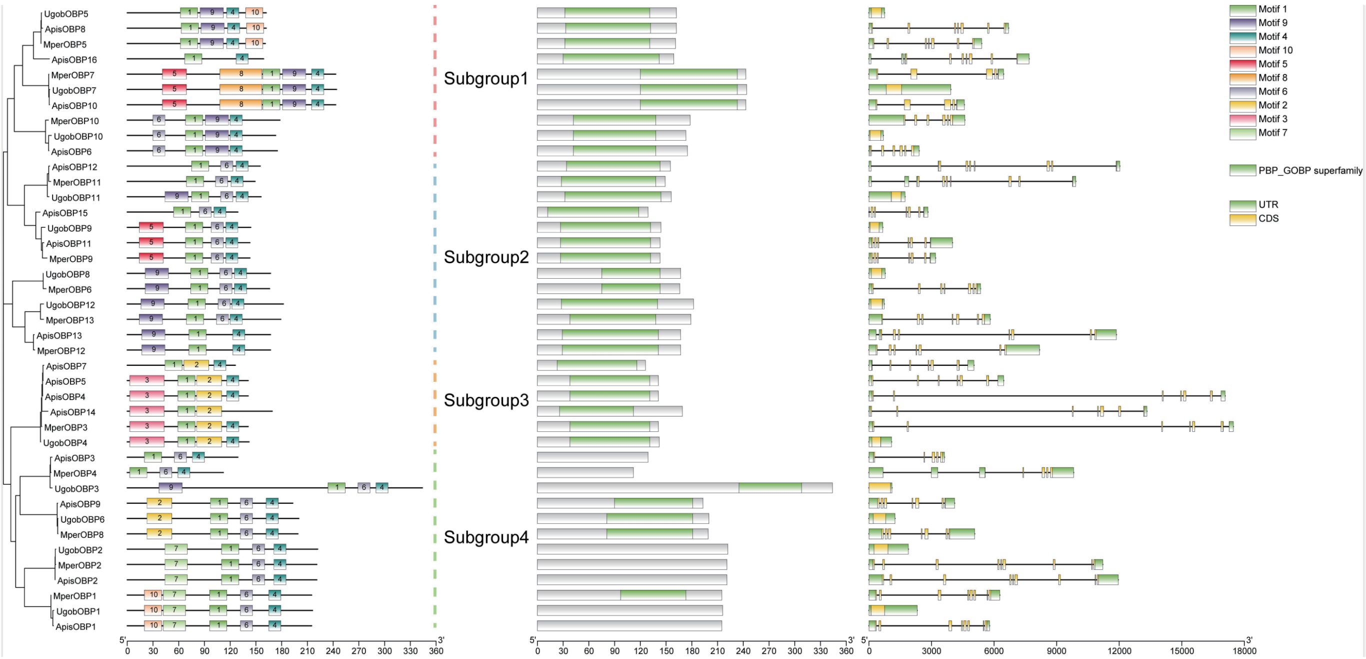

Figure S5: Analysis of phylogenetic relationships, protein domains, and gene structures of OBPs

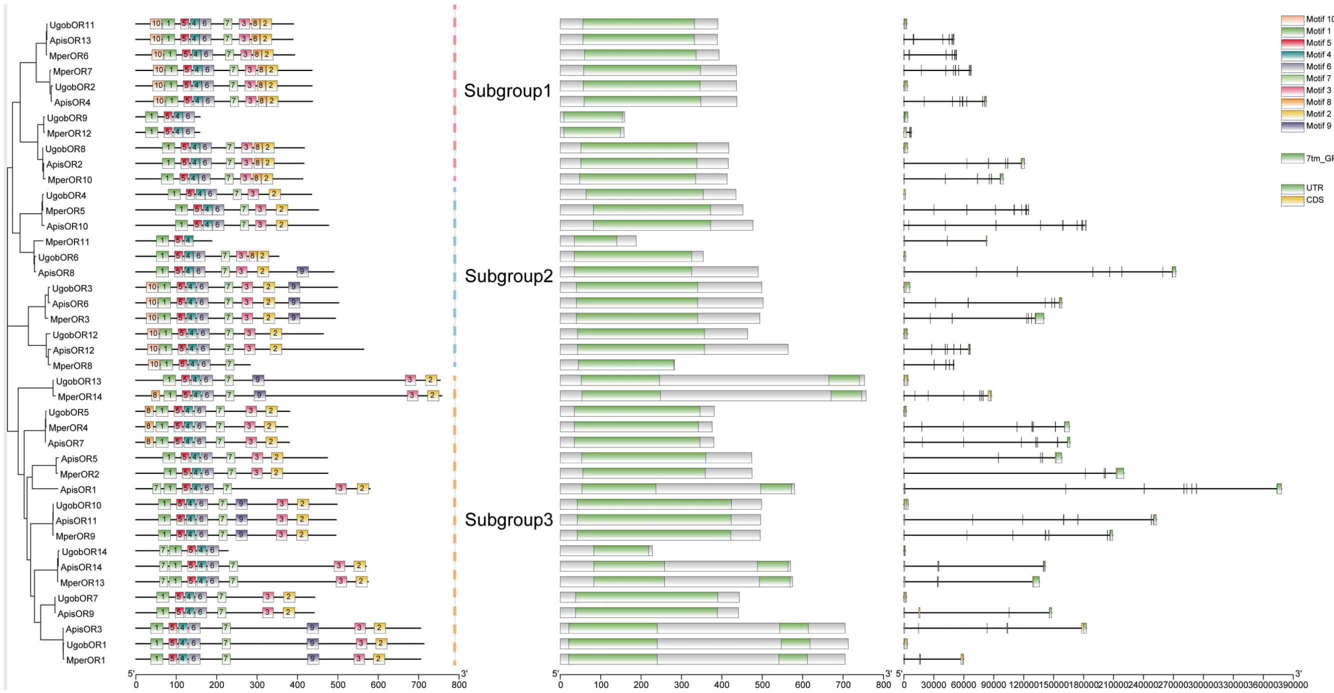

Figure S6: Analysis of phylogenetic relationships, protein domains, and gene structures of ORs

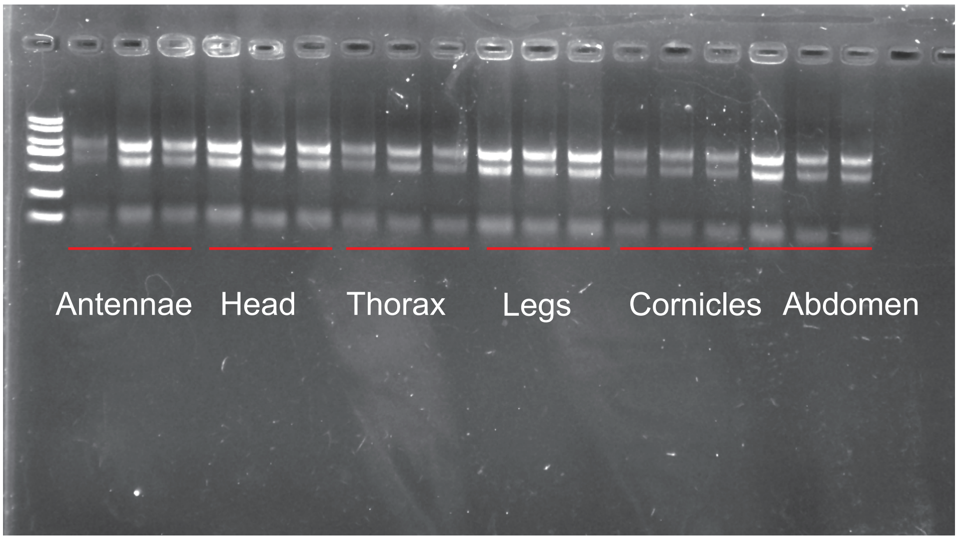

Figure S7: Agarose gel electrophoresis analysis of PCR products for tissue-specific expression validation
